# Elucidating sole application of farmyard manure and blended NPSB fertilizer effects on soil properties at Bench Shako and West Omo zone, South West Ethiopia

**DOI:** 10.1016/j.heliyon.2023.e22908

**Published:** 2023-12-01

**Authors:** Tesfaye Lishan, Fessha Alemu

**Affiliations:** Department of Plant Science, College of Agriculture and Natural Resource, Mizan Tepi University, Ethiopia

**Keywords:** Farm yard manure, Soil properties, Sole application, Mineral fertilizer

## Abstract

Soil nutrient depletion is the main problem in Ethiopia for agriculture sector. Comparison of organic and inorganic source of fertilizer is needed for soil physicochemical properties improvement. Hence, the current study was investigated to investigate the sole application of farmyard manure and blended NPSB fertilizer on soil properties. For this investigation, farm yard manure at three level (0,5, and 10 t ha-1) and NPSB at four level (0, 50, 100, and 150 kg ha-1). All collected data were analysed by GenStat software. Sole application of farm yard manure significantly improves soil bulk density ((1.23 gcm^3^ to 1.11 gcm^3^ at kuja Guraferda, and 2.22 gcm^3^ to 1.12 g/cm3 deka Maenit-Goldia) and soil moisture content from 12.14 % to 33.79 % at kuja, and 11.88 % to 36.01%at deka). More over application of farm yard manure improve soil pH (6.15) organic carbon (6.20 mg/kg) available phosphorus (18.94 mg/kg, total nitrogen (0.32), sulfur (18.27), boron, exchangeable base, cation exchange capacity, micronutrients (copper, manganese & zinc). Application of NPSB fertilizer improves only, total nitrogen, available phosphorus, sulfur and boron. Application of Farm yard manure significantly improve soil physicochemical properties than NPSB mineral fertilizer in comparison. Therefore, application of farm yard manure is more important than mineral NPSB fertilizer for sustainable soil physical and chemical properties improvement from soil management aspect. Thus, application farmyard manure at 10 t/ha is recommended for the studied soil at West Omo zone Maenit-Goldia district, Deka kebele, and Bench-Skako zone, Guraferda district at Koyi kebele, south west people regional state, South West Ethiopia*.*

## 1 Introduction

Soil degradation due to the addition of chemical fertilizer is among the major factors affecting soil physicochemical properties. In Ethiopia, soil degradation and nutrient depletion have gradually increased and caused serious drawbacks to agricultural productivity. The management of agricultural systems influences soil physical and chemical properties through fertilization, tillage, crop residue management, and other practices [1,2].

In order to get a high crop yield, chemical fertilizer has been applied repeatedly, which has worsened soil degradation, increased soil acidity, and affected soil health. In order to maintain soil's physicochemical properties in a sustainable manner as an alternative to chemical fertilizers, organically based natural fertilizer is a viable option [2]. Currently, the world places emphasis on the utilization of organic sources of fertilizer to enhance and sophisticate organic farming for the best utilization and management of soil resources in sustainable ways [3]. Organic fertilizer improves soil physical, chemical, and biological properties in sustainable ways when compared with mineral fertilizers. Manure and composted organic wastes are known and applied for soil restoration, soil nutrient improvement, and environmental protection through sustainable soil management [1]. Farmyard manure has some advantages for use as organic fertilizer because it is a source of organic carbon that microbes require to enhance their growth promotion activities under drought condition[2] (. Organic materials are able to keep nutrients in soils and improve soil structure and fertility [3]

Organic fertilizers also keep soil healthy, increase plant resistance to hard environmental conditions, and improve their properties [1][4]; [5]. Organic agricultural practices aim to enhance biodiversity, biological cycles, and soil biological activity to produce optimal natural systems that are socially, ecologically, and economically sustainable. The use of these fertilizers at the appropriate ratio may be valuable in increasing crop yields and keeping soil healthy. Farmyard manure application improves soil carbon stock, aggregate stability, soil pH [6], and decreases nutrient loss [1]. The declining of soil nutrients, the deterioration of soil physicochemical properties, and soil acidity problems and crop yield reduction are looking like sustainable soil management problems. Sesame crop production is facing various problems such as lack of appropriate agronomic practice, low soil fertility and high costs of chemical fertilizers. In Ethiopia, soil degradation and nutrient depletion gradually increased in area and magnitude and have become serious threats to agricultural productivity. The majority of soils in south western Ethiopia are deficient in macronutrients and micronutrients [7]). In south western part of Ethiopia, application of mineral/chemical fertilizer trial has been done so far for the imprudent soil nutrient and crop production enhancement. However, the trial was conducted for NPL, urea, currently on some blended fertilizer like NPS; but not addressed sole application effects of farm yard manure (FYM) and newly released blended NPSB fertilizer soil fertility management.

To overcome the problem, sustainable soil management by using organic sources of soil fertilizer is an optional approach. Therefore, the present trial was conducted to evaluate the effects of sole application of farmyard manure and blended NPSB synthesized fertilizer on soil physicochemical properties. Bench Shako and Maenit-Goldia zone, South Western Peoples Regional State, South Western, Ethiopia.

## 2 Materials and methods

### 2.1 Description of the study area

The experiment was conducted over two locations namely Guraferda from Bench shako zone and Maenit-Goldia from west Omo zone, south western Ethiopia. **Guraferda district** was located in Bench-Shako zone, south west people regional state south western Ethiopia. Guraferda district was located 62 Km far from Mizan Teferi (Zonal town). Guraferda site is geographically positioned between 6° 49′ 33″N latitude and 35° 07′ 03″E longitude respectively. The study was found 900 m.a.s.l. The areas are categorized as sub-humid climate since they are located in sub-tropics. The average rainfall in the area was 1100 mm and minimum and maximum temperatures are 17.46 and 29.83 °C respectively. The dominant crops in the study areas are rice, maize, haricot bean and cash crop such sesame as oil crop, fruit such as mango, avocado and different spices crops. Soil of Guraferda was characterized by clay loam texture; and *orthric* Acrisol and dystric nitosol were the dominant soil types. **Maenit-Goldia district** was situated in West Omo one, south wester people reginal state, South western Ethiopia. Maenit-Goldia district was located 105 Km far from Mizan Teferi. Maenit-Goldia district was geographically positioned, between 6° 58′ 06 ″ N latitude and 35° 25′ 0 2° “E longitude respectively. The study was found 1238 m.a.s.l. The average rainfall in the area was 1100 mm. And minimum and maximum temperatures are 15.78 and 27.00 °C respectively. The dominant crops in the study areas are rice, maize, haricot bean and cash crop such sesame as oil crop, fruit such as mango, avocado and different spices crops. Soil of Maenit-Goldia district was characterized by clay texture; and soil type of the study area was dystric Nitosol.

### 2.2 Experimental materials, treatments, experimental design and procedures

Farm yard manure was used as source of organic fertilizer and NPSB was used as a source Chemical fertilizer. Factorial arrangements of three levels of (0t, 5t and 10t FYM ha^−1^) farmyard manure and four levels of (0 Kg, 50 Kg, 100 Kg, and 150) kgha^−1^ NPSB fertilizes laid out in randomized complete block design (RCBD) with three replications.

Two pre-sowing and 24 post-harvest composite soil samples were collected from each district of experimental field before sowing and after-harvesting respectively., Undisturbed soil sample was collected using core sampler for soil moisture content and bulk density determination and disturbed soil was collected for total nitrogen, available phosphorus, Sulfur, soil pH, organic carbon, cation exchange capacity, exchangeable bases and micronutrient analysis by using Auger. The soil sample was air dried and packed in a polythene bag, labelled and transported to HORTCOOP national soil Laboratory at Debrezeyt and analysed for selected soil physic chemical properties.

**Soil Physical properties** were analysed for Soil particle size (texture), Bulk density and soil moisture content. Soil bulk density was determined by oven drying method. Particle size distribution or Textural class was determined by Bouyoucos hydrometer. Moisture content will be determined by measuring the weight of the soil, before and after oven dried for 24 h at 105 °C as described.

**Soil Chemical parameters** like soil pH, soil organic carbon (SOC), total nitrogen (TN), available-phosphorus and sulfur, exchangeable bases, cation exchange capacity (CEC), ex-acidity and micronutrients were analysed by respected methods. The soil pH of the soil was measured potentiometrically using a digital pH meter. Soil organic carbon: was determined by Walkley and Black (1934) methods. Total nitrogen was analysed by using the Kjeldahl digestion method. Available phosphorus was determined by Olsen method. Available Sulfur was determined in the soil extract by the turbidity method. Exchangeable bases (Ca^+2^, Mg^+2^ and Na^+^) were determined Mehlich-3 (1984). Cation exchange capacity was determined by Ammonium Acetate Method. Micronutrients and Boron were extracted Mehlich-3 (1984) methods.

### 2.3 Data analysis

All composed data were analysed using GenStat software 18th edition. For significant treatment effects, the mean separation was made using the Least Significance Difference (LSD) test at 5 % level significance.

## 3 Result and discussion

### 3.1 Sole effects of farm yard manure and NPSB fertilizer on soil bulk density and moisture content

The analysis of variance showed that applying Farm yard manure improved soil bulk density and moisture content at both districts. Application of farm yard manure significantly (p < 0.001) improve soil bulk density and soil moisture contents studied soil. However, application of chemical fertilizer in the form of NPSB was not significantly influenced both soil bulk density and soil moisture content at both studied soil (Table 1). Accordingly, the lowest soil bulk density (1.11 g/cm3 and 1.12 g/cm3) were recorded at Kuja kebele Guraferda district and Deka Kebele Maenit-Goldia district from the application of 10 t/ha FYM. While the maximum soil bulk density was recorded from the application of farm yard manure at 10 t/ha (Table 2) and the highest value (1.23 and 2.22 g/cm3) and the lowest (1.11 and 1.12 g/cm3) of bulk density were recorded from controlled treatment and application of FYM at 10 t/ha at both locations respectively. Likewise, the highest (33.79 % and 36.01 %) were recorded from the application of FYM at 10 t/ha at both locations respectively. While the minim moisture (12.14%and 11.88 %) was recorded from controlled plot at both studied locations.

**Table 1:** Pre-sowing soil properties at Kuja kebele Guraferda and Deka kebele Bachuma Districts.

Soil parameters		
	Kuja kebele Guraferda	Deka kebele maenit goldia
Soil physical parameters		
Bulk density	1.32	1.31
Soil moisture content	13.21	12.20
Texture	Clay loam	Clay
**Soil Chemical parameters**		
PH -H_2_O	5.48	5.54
Ca (mgkg^−1)^	3183.22	3259.84
Mg (mgkg^−1)^	527.00	504.07
K (mgkg^−1)^	218.30	437.51
Na (mgkg^−1)^	42.50	20.93
Av. P (mgkg^−1)^	12.26	15.99
S (mgkg^−1)^	6.39	7.08
B (mgkg^−1)^	0.28	0.38
Total Nitrogen TN (%)	0.23	0.25
Organic carbon OC (%)	3.93	3.31
CEC meq/100gsoil	24.62	25.32
Cu (mgkg^−1)^	2.62	2.01
Mn (mgkg^−1)^	28.40	37.25
Zn (mgkg^−1)^	3.27	3.97

**Table 2:** Effects of farm yard manure and chemical fertilizer (NPSB) on soil bulk density and moisture content.

Kuja Kebele, Guraferda District			Deka kebele. Maenit-Goldia District	
**NPSB (kg/ha**)	Bulk density (gcm^−3^)	Moisture (%)	Bulk density (gcm^−3^)	Moisture (%)
0	1.23	11.11	1.24	10.01
50	1.22	12.70	1.23	10.19
100	1.23	12.11	1.24	10.18
150	1.22	12.80	1.23	11.72
**Lsd**_**0.05**_	**0.01451**	**1.329**	**0.01258**	**1.519**
***P*-value**	**ns**	**ns**	**ns**	**ns**
**CV (%)**	**1.3**	**7.2**	**1.1**	**6.2**
**FYM (t/ha)**				
0	1.24^a^	12.14^c^	1.23^a^	11.88^c^
5	1.14^b^	22.31^b^	1.18^b^	27.41^b^
10	1.11^c^	33.79^a^	1.12^c^	36.01^a^
**Lsd**_**0.05**_	**0.01256**	**1.064**	**0.0109**	**1.315**
***P*-value**	**	**	**	**
**CV (%**	**1.3**	**6.1**	**1.1**	**6.2**

**Note**: NS: not significance difference; **: highly significant; L.S.D: least significance difference; CV: coefficient of variation.

Bulk density is used to characterize soil compaction, which influences the structural functions and characteristics of soils. Applications of farmyard manure are more responsive than mineral fertilizer NPSB. The improvements in soil bulk density and soil moisture content in the studied soil attributed to the organic matter content in the farmyard manure released through decomposition. Soil bulk density was highest in pre-sowing, controlled and NPSB treated treatment due to low in organic matter content of soil in both studied soils as compared to Farm yard manure treated treatment in the studied soil of kuja Guraferda and deka Maenit-Goldia districts.

The current study agreed with the finding by Ref. [8] that the application of organic sources of fertilizer like animal manure improves soil physical stability through aggregation, enhances water holding capacity, and reduces bulk density. The increasing application of organic sources of fertilizers increases organic matter content, which results in greater total porosity, lowers soil bulk density, and improves soil moisture contents [9]). Similar findings by [8] determined that applications of manure contributed to improving soil structure and soil moisture retention. Similarly,[9] explained that the addition of animal manure improves aggregate stability and soil bulk density.

Similar findings conducted in the Amhara region at Fogera by [9]) showed that the use of farmyard manure expressively increased soil bulk density and water holding capacity [9]. stated that the enhancement of soil moisture contents in response to the addition of manure is due to amended soil structure and moisture stability as well as increased water storage pores. The addition of farmyard manure and other organic sources of fertilizer has high responsive characteristics in improving and maintaining soil physico-chemical properties in sustainable ways when compared with mineral fertilizers.

Effects of Sole application of FYM and NPSB on soil PH, organic carbon, Total nitrogen, av. Phosphorus and Sulfur, Sole application of farmyard manure showed significant (p < 0.001) effects on some tested soil chemical parameters such as; soil PH, organic carbon, total nitrogen, available phosphorus, and available sulfur at both locations (Table 2). Similarly, application of mineral fertilizer NPSB showed significant (p < 0.001) effects on total nitrogen, available phosphorus, boron, and sulfur, but didn't show significant effects on soil PH, total nitrogen, soil carbon, or available potassium at both studied locations (Table 3).

**Table 3:** Main effects of NPSB and FYM on soil PH, organic carbon, total N, available phosphorus and Sulfur.

Kuja kebele soil Guraferda Districts					
NPSB (kg/ha)	PH	OC (%)	TN (%)	Av.P (mg/kg)	S (mgkg)
0	5.42	2.25	0.21^b^	8.49^b^	5.56^c^
50	5.49	3.23	0.21^b^	17.53^a^	12.81^b^
100	5.27	3.25	0.22^b^	17.95^a^	14.46^a^
150	5.20	3.23	0.25^a^	18.17^a^	15.01^a^
**LSD**_**0.05**_	**0.225**	**0.251**	**0.012**	**0.919**	**0.795**
***P*-value**	**ns**	**ns**	**	**	**
**CV (%)**	**4.2**	**5.9**	**4.7**	**5.4**	**6.0**
0	4.86^c^	2.21^c^	0.13^c^	8.36^c^	5.78^c^
5	5.96^b^	4.20^b^	0.26^b^	17.78^b^	13.94^b^
10	6.15^a^	6.20^a^	0.32^a^	18.94^a^	18.27^a^
**LSD**_**0.05**_ ***P*-value**	0.195 **	0.194 **	0.010 **	0.796 **	0.689 **
**CV (%)**	**4.2**	**5.9**	**4.7**	**5.4**	**6.0**
Deka Kebele soil Maenit-Goldia District					
0	5.24	2.14	0.20^bc^	8.67c	4.53^b^
50	5.21	3.13	0.21^ab^	15.62^ab^	11.68^a^
100	5.10	3.13	0.22^a^	16.39^a^	12.69^a^
150	5.21	3.14	0.23^a^	17.24^a^	12.79^a^
**LSD**_**0.05**_	**0.097**	**0.120**	0.019	1.1371	**1.280**
***P*-value**	**ns**	**ns**	**	**	*
**CV (%)**	**1.7**	**3.4**	7.5	8.9	**11**
0	5.12	2.31	0.21	7.01	5.70
5	5.96	4.58	0.29	16.66	13.12
10	6.41	5.80	0.30	18.52	17.95
**LSD**_**0.05**_ ***P*-value**	0.084 **	0.104 **	0.016 **	1.187 **	1.109 **
**CV (%)**	**1.7**	**3.4**	7.5	8.9	**11**

**Note:**NS: no significance difference between treatment means, **: highly significance difference b/n treatment means; *: significance difference b/n treatments mean.

Accordingly, the highest values of soil pH (6.15 and 6.41) were recorded from the higher level of farmyard manure at 10 t/ha at both locations, respectively, while the lowest values of soil pH (4.86 and 5.12) were recorded from the controlled plot (Table 3). Similarly, the highest soil organic carbon values (6.20 % and 5.80 %) were recorded from 10 t/ha of farmyard manure, while the lowest values (3.21 % and 3.14 %) were recorded from controlled or untreated plots at both Kuja kebele Guraferda and Deka kebele Maenit-Goldia district, respectively.

The highest values of total nitrogen (0.32 % and 0.13 %) and (0.25 % and 0.21 %) were recorded from the application of farmyard manure at 10 t/ha and NPSB at 150 kg ha-1 at both studied locations, respectively (Table 3). Soil total nitrogen in the study area was improved both by the application of farmyard manure and NPSB fertilizer. Likewise, application of farmyard manure improves soil available phosphorus from (8.36 mg/kg to 18.94 mg/kg at deka Guraferda district, and from 8.67 mg/kg to 17.24 mg/kg at deka kebele Maenit-Goldia districts (Table 3). Similarly, application of NPSB blended fertilizer also increased available phosphorus from 8.49 mg/kg to 18.17 mg/kg at kuja Guraferda district, and from 8.67 mg/kg to 17.24 mg/kg were recorded from studied soil of deka Maenit-Goldia districts. Sulfur also improved by the applications of farmyard manure and NPSB fertilizer (10 t/ha FYM and 150 kg NPSB/ha (Table 3).

**Soil** pH is among the main soil chemical properties attributes which determine availability of nutrients and other related functions. Farmyard manure application had a greater impact on soil pH than NPSB fertilizer application, which couldn't demonstrate any appreciable changes in the two soils under study. On the other hand, increasing the application of NPSB chemical fertilizer lowers soil pH, which has an impact on the availability of nutrients and is detrimental to plant growth. Similar results were found by Ref. [6]; who found that applying animal manure increased soil carbon content while and maintaining soil pH. similarly [8], reported that applying synthetic fertilizer decreased soil pH.

**Soil organic carbon and total nitrogen** was increased with increasing farm yard manure use rate from 0 t ha-1 to 10 t ha-1; whereas, NPSB addition to soil did not improved or increased soil organic carbon and total nitrogen both at Kuja kebele Guraferda and Deka kebele Maenit-Goldia's districts soil. We applied well decomposed farm yard manure under suitable conditions which contain higher dose of nutrients and easily mixed with soil when applied.

According to Ref. [10]; soil organic carbon and total nitrogen play a significant role in the ecosystem's soil processes. Farm yard manure's organic matter content appears as stable organic compounds upon decomposition [11], and its long-term use as a source of soil inputs may be the key to maintaining the high level of soil organic carbon. The differences in the organic carbon content of amended soils can be attributed to the chemical composition, and decomposability of the residues applied.

Our experiment's results were in line with a study by [10], which found that applying manure to the soil considerably enhanced the amount of soil organic carbon compared to using mineral fertilizer alone. Similar to this, Dunjena et al. (2012) noted that soil organic carbon increases when manure application rates rise. When organic manure is applied, soil characteristics, nutrient availability, and the magnitude or quantity of soil organic carbon are all significantly improved [12]. Because organic manure decomposes during application, Walker and Bernal (2008) found that applying it considerably boosted the soil's nitrogen content. Similar research by Tilahun et al. (2013) in Fogera in the Amhara region revealed that the use of farmyard manure significantly boosted soil organic carbon.

**Available phosphorus** is enhanced by the application of organic and inorganic fertilizer in sustainable way by organic and for recurrent or seasonal by mineral fertilizers. The results showed that applying farmyard manure and NPSB mineral fertilizer increased the amount of phosphorus that was accessible in the soil in both of the study locations. This may indicate that FYM application has contributed to increasing and maintaining soil phosphorus levels; similarly, applied NPSB mineral fertilizer also contains phosphorus that is readily available but has been blended to increase/add phosphorus to the soil. When compared to mineral fertilizer NPSB, application of farmyard manure enhances more (Table 3). Application of organic manure as a main fertilizer or, alternatively, mineral fertilizer increases the amount of phosphorus that is readily available in the soil. Similar research by Ali et al. (2007) found that the addition of farmyard manure increased the amount of soil-available phosphorus and postponed the fixation of soil phosphorus by organic anions created during farmyard manure decomposition. Additionally, study by Gidago et al. (2011) at Areka, south-western Ethiopia, showed that applying mineral fertilizer with a higher proportion of phosphorus content improved the amount of phosphorus that was available in the soil. By chelating soil Fe and Al with organic acids generated during decomposition, organic fertilizer complements phosphorus and improves the efficiency of applied phosphorus [13].

**Sulfur** is significantly improve by sole applications of farm yard manure and blended NPSB fertilizer. Farmyard manure and blended NPSB fertilizer applied alone considerably reduce sulfur levels (Table 3). Compared to the control treatment, the application of farm hard manure alone led to an increase in soil sulfur. Additionally, applying farm yard manure increases sulfur concentrations relative to blended NPSB [14]. made a similar discovery, reporting that the usage of farmyard manure increases the availability of sulfur for plants because just applying farmyard manure releases more nutrients through decomposition. This result agreed with that of Tewolde et al. (2020), who noted a significant increase in soil sulfur as a result of the highest rate of NPSB fertilizer application.

## 4 Effects of sole application of FYM and NPSB on soil exchangeable base and CEC

The present study showed the applications organic sources of fertilizer improve exchangeable base and cation exchange capacity. Accordingly, analysis of variance indicated that the application of farmyard manure significantly (p < 0.001) improve soil exchangeable base and cation exchange capacity; however, the application of mineral fertilizer NPSB could not significantly (p > 0.05) affect soil exchangeable base and CEC of soil except sodium (Table 4). Accordingly, exchangeable potassium also influenced by the application of farm yard manure at 5t ha-1. Accordingly, the highest (337.08 mg/kg and 343.32 mg/kg) were recorded from the application of farm yard manure at 10 t/ha in both studied soil of kuja, Guraferda and deka Maenit-Goldia district respectively. While the lowest (291.14 mg/kg and 294.08 mg/kg) were recorded from controlled treatment at both studied soil.

**Table 4:** Effect of Sole application of farm yard manure and chemical NPSB fertilizer on soil exchangeable base and cation exchange capacity.

Kuja kebele soil					
NPSB (kg ha−1)	K (mg kg−1)	Ca (mg kg−1)	Mg (mg kg−1)	Na (mg kg−1)	CEC (mg kg^−1^)
0	294.41	2141	448.0	47.72	16.1
50	291.68	2140	442.4	49.47	19.88
100	290.67	2130	440.8	47.10	18.94
150	290.47	2139	440.8	50.31	19.84
**LSD**_**0.05**_	**4.917**	**43.9**	**10.33**	**3.22**	**2.744**
***P*-value**	**ns**	**ns**	**ns**	**ns**	**ns**
**CV (%)**	**2.8**	**1.3**	**3.5**	**4.7**	**9.4**
**FYM (t ha**^**−**^**^1^)**					
0	291.14^c^	2181^c^	449.0^c^	40.02^c^	14.71^c^
5	315.71^b^	3432^b^	515.6^b^	54.68^b^	29.54^b^
10	337.08^a^	3845^a^	610.6^a^	60.24^a^	35.43^a^
**LSD**_**0.05**_	**6.856**	**38.0**	**15.87**	**2.050**	**2.377**
***P*-value**	**	**	**	**	**
**CV (%)**	**2.8**	**1.3**	**3.5**	**4.7**	**9.4**
**Deka kebele soil**					
0	294.01	3234.2	460.79	20.30	20.38
50	288.84	3263.4	452.77	21.44	19.29
100	278.27	3248.9	452.21	21.07	19.15
150	290.57	3228.0	450.82	22.35	20.76
**LSD0.05**	**12.042**	**93.55**	**8.643**	**2.296**	**2.184**
**P-value**	**ns**	**ns**	**ns**	**ns**	**ns**
**CV (%)**	**4.3**	**2.7**	**1.6**	**2.3**	**7.5**
**FYM (t ha−1)**					
0	294.08^c^	2211.3^c^	410.22^c^	50.65^c^	16.70^c^
5	386.36^b^	3412.2^b^	532.02^b^	57.61^b^	24.46^b^
10	343.32^a^	3987.5^a^	620.20^a^	65.35^a^	33.53^a^
**LSD**_**0.05**_	**10.428**	**81.016**	**7.485**	**1.148**	**1.891**
***P*-value**	**	**	**	**	**
**CV (%)**	**4.3**	**2.7**	**1.6**	**2.3**	**7.5**

Note:**: highly significance difference b/n treatment mean; *: significance difference b/n treatment mean.

Similarly, the highest calcium (3845 mg/kg & 3987.5 mg/kg) were recorded from the application of farm yard manure at the rate of 10 t/ha at both studied soil of kuja and deka; while the minim value (2181 mg/kg & 2211 mg/kg) were recorded from controlled treatment at both studied soils of kuja Guraferda and deka Maenit-Goldia (Table 4).

Similarly, application of farmyard manure increases the exchangeable magnesium, sodium, and cation exchange capacity with increasing use rates. Exchangeable magnesium increased from 449 mg/kg to 610.6 mg/kg by the application of farmyard manure from 0 t/ha to 10 t/ha at kuja soil area; and it increased/enhanced from 410.22 mg/kg to 620.20 mg/kg by the application of 0 t/ha to 10 t/ha of farm yard manure at deka kebele Maenit-Goldia district.

Sodium also increased from 40.02 mg/kg from controlled plot to 60.24 mg/kg which recorded from 10 t farmyard manure/ha) at the studied soil of kuja Guraferda districts. Similarly, sodium influenced by the application of FYM at 10 t/ha at studied soil of Deka, Maenit-Goldia district. Cation exchange capacity improved (*14.*71 mg/kg *to 35.*43 mg/kg*, and from 16.*70 mg/kg *to 33.*53 mg/kg) by the application of farm yard manure at the highest use rate 10 t/ha both at studied soil of kuja Guraferda and Deka Maenit-Goldia district (Table 4).

The variation between the level of farm yard manure applications on CEC might be accredited to the existence of organic matter in the soil, in addition to the applied farm yard manure which might be improved and lead to more CEC of soil.

The difference in the amount of applied farm yard manure and soil CEC may be attributed to the presence of organic matter in the soil as well as the applied farm yard manure, which may improve soil CEC. As a source of CEC and soil buffering, farm yard manure application served as a reservoir for several critical components. The use of farmyard manure may have improved the soil's cation exchange capacity and exchangeable base, which may be responsible for the improvements in exchangeable calcium and magnesium. The results of the present study are consistent with those of Vanlauwe et al. (2015), who found that the use of organic fertilizers rich in organic matter has a strong potential to improve exchangeable base and cation levels.

Application of organic sources of fertilizer rich in organic matter has a high tendency to improve exchangeable base and cation exchange capacity in particular and soil physico-chemical properties in general, according to the current study, which is in line with the findings by Vanlauwe et al. (2015). According to Ref. [15]; clay and organic matter content are the two key chemical characteristics of soil that influence cation exchange capacity. The amount of organic matter included in organic sources of fertilizers affects the physical, chemical, and Cation Exchange Capacity (CEC) of the soil surface, as well as the stability of soil aggregates [16]. The increase in CEC has also been linked to more negatively charged surfaces in the organic matter. Similarly, the increment of CEC has been accredited to increased negatively charged surfaces present in the organic matter of the organic soil inputs [17].

Increases in the soil cation exchange capacity and buffering capacity of soil have also been detected and ascribed to the abundant humic substances present in the applied compost [18]. The positive effects of sole application of alterations of soil physical and chemical properties were cheap, extremely available organic fertilizer source like farm yard manure and compost [19]. The result of this experiment agreed with the previous reports by Ref. [16] determined that the use of manure improves the soil by the formation of clay humic complexes which rise the soil adsorbent volume of exchangeable bases (calcium, magnesium and potassium) and improve microbial activity involved in mineralization process in the soil surroundings. Similarly, the recent findings in Nigeria by Ref. [20] determined that post-harvesting soil analysis indicated increased in animal manures intensely amended in soil acidity, and nutrient availability and increased in exchangeable calcium, magnesium, potassium due to application of manures. Likewise, [21]; application of farm yard manure increases exchangeable base.

### 4.1 Effects of sole application of farm yard manure and blended NPSB on soil micro nutrients

Analysis of variance showed application of farm yard manure significantly (P < 0.001) enhanced soil micronutrients (boron, copper, manganese & zinc); however, application of NPSB was not significantly (p > 0.05) influenced soil micronutrients except iron contents (Table 5). Hence, the highest value of copper, manganese and zinc (2.35mgkg,17.35 mg/kg & 2.76 mg/kg) was obtained from the application of farm yard manure at use rate of 10 t ha^−1^, while the lowest value of Copper, Manganese and Zinc (2.19 mg/kg, 13.17 mg/kg, & 2.12 mg/kg) were obtained from unfertilized or controlled plot. Similarly, the highest value of copper, manganese and zinc (2.65 mg/kg, 15.18 mg/kg & 2.79 mg/kg) was obtained from the application of farm yard manure at use rate of 10 t ha^−1^, while the lowest value of Copper, Manganese and Zinc (2.01 mg/kg, 13.51mgkg, & 2.20 mg/kg) were obtained from controlled treatment at Deka Kebele (Table 5). Application of FYM and NPSB fertilizer improve soil boron. Accordingly, the highest value (0.32 mg/kg &0.29 mg/kg) were recorded from the application of FYM at 10 t/ha and 150 kg NPSB/ha at Kuja Guraferda district. Similarly, the highest (0.35 mg/kg and 0.32 mg/kg) were recorded from the highest application rate of FYM and NPSB at Deka kebele Maenit-Goldia districts (Table 5).

**Table 5:** Main effects of NPSB and FYM on Soil Micronutrients.

Kuja kebele Guraferda District				
**NPSB (kg/ha)**	**B(mg/kg)**	**CU (mg/kg)**	**Mn (mg/kg)**	**Zn (mg/kg)**
0	0.25^c^	2.11	16.4	2.17
50	0.26^b^	2.13	14.54	2.17
100	0.29^a^	2.14	14.22	2.15
150	0.29^a^	2.15	15.14	2.13
**LSD**_**0.05**_	**0.003**	**0.066**	**1.827**	**0.053**
***P*-value**	*	**ns**	**ns**	**ns**
**CV (%)**	**1.1**	**3.0**	**12.4**	**2.8**
**FYM (t ha**^**−**^**^1^)**				
0	0.22^c^	2.19^c^	13.17^b^	2.12^c^
5	0.29^b^	2.28^b^	14.70^b^	2.36^b^
10	0.32^a^	2.35^a^	17.35^a^	2.76^a^
**LSD**_**0.05**_	**0.003**	**0.057**	**1.582**	**0.072**
***P*-value**	**	**	**	**
**CV (%)**	**1.1**	**3.0**	**12.4**	**3.5**
**Deka Kebele Maenit-Goldia district**				
**NPSB (kg ha-1)**				
0	0.25^c^	2.17	16.19	2.10
50	0.28^ab^	2.15	15.66	2.15
100	0.30^a^	2.13	14.44	2.14
150	0.32^a^	2.17	14.11	2.17
**LSD**_**0.05**_	**0.022**	**0.338**	**2.171**	**0.211**
***P*-value**	**	**ns**	**ns**	**ns**
**CV (%)**	**7.6**	**15**	**14.7**	**4.5**
**FYM (t ha**^**−**^**^1^)**				
0	0.24c	2.01^b^	13.51^b^	2.20^c^
5	0.27b	2.26^b^	16.61^a^	2.63^b^
10	0.35a	2.65^a^	15.18^a^	2.79^a^
**LSD**_**0.05**_	**0.019**	**0.293**	**1.88**	**0.096**
***P*-value**	**	**	**	**
**CV (%)**	**7.6**	**15**	**14.7**	**4.5**

**Note:**NS: no significance difference b/n treatment means; **: highly significance difference b/n treatment mean; *: significance difference b/n treatment mean.

According to the data presented in (Table 5) for micronutrients (Cu, Mn & Zn) showed that the value of micronutrient contents significantly improved by the application of farm yard manure over blended NPSB fertilizer and controlled treatment over the studied two locations.

When compared to blended NPSB fertilizer, farmyard manure has the capacity to add micronutrients to the soil, even in small concentrations. Farmyard manure has the ability to deliver micronutrients to the soil, even in small amounts, when compared to blended NPSB fertilizer. The current study's findings highlight the value of farmyard manure in boosting soil micronutrients. Boron is an essential component that helps plants use macronutrients more effectively and transport photons from the source to the sink more effectively while they are growing. The tested soil's boron concentration is markedly increased by the addition of mixed NPSB fertilizer and lone farm yard manure. The rise in boron content could be attributed to the residual NPSB and farmyard manure that were put to the soil. The researched area's soil lacks boron, hence blended fertilizers containing boron are suggested, according to Ref. [7]. According to a study by [22], the usage of blended NPSB fertilizer reduces the availability of boron deficiency in the Bedelle province of south-western Ethiopia. The increases in soil micronutrient levels could be attributed to a variety of factors, including the efficiency of applied fertilizers, their composition, and the rate at which manure additions break down. One of the organic soil inputs necessary to improve the physical, chemical, and biological properties of the soil as well as its fertility is the use of farmyard manure. Farmyard manure improves soil micronutrient because of its high amount of macro- and micronutrients, which are released after decomposition. Applying farm yard waste [22], also came to the same outcome. According to Ref. [23]; although in modest amounts compared to mineral fertilizers, manures have the ability to add macro- and micronutrients to the soil for plant growth. According to [24], the addition of organic manures resulted in a decrease in the redox potential of the soil, which increased the release of soil micronutrients in accessible form compared to the application of chemical fertilizers alone. Because they created a favourable environment for oxidation and reduction regimes and the lowering of soil pH, organic manures were a major factor in the release of Zn, Cu, Fe, and Mn in the rice-wheat system [25].

## 5 Conclusion

Organic Source of fertilizer is known by improving soil physical, chemical and biological properties in sustainable ways when compared with mineral source of fertilizers. Application of chemical fertilizers are known by their easily available manner for plants. But, repeatedly application of chemical fertilizer significantly affects physical and chemical properties of soil, special highly rainfall or acid dominated area like south western Ethiopia where current experiment conducted. On the other hands, application of organic fertilizer like FYM has recognized for their role in improving soil physico-chemical and biological properties in sustainable ways. Thus, the current finding indicated that application of Blended NPSB mineral fertilizer improve only available phosphorus, sulfur, boron; but, not give response for exchangeable base, cation exchange capacity, soil micronutrients and minimize soil PH. In contrary, application of FYM significantly improve soil physical, chemical properties like, soil PH, available phosphorus, total nitrogen, organic carbon, Sulfur content, exchangeable base, cation exchange capacity, and soil micronutrients. South western Ethiopia is characterized by highly rainfall area and is vulnerable for nutrient leaching and soil erosion. So, application organic source of fertilizer like FYM at 10 t/ha is recommendable for nitiso at West Omo zone Maenit-Goldia district, Deka kebele, and for acriso and nitosol of Bench-Skako zone, Guraferda district at Kuja kebele, south west people regional state, South West Ethiopia.

## Funding

The authors declare that there is no specific funding from any fundraising organizations.

## Data availability

Data will be made available from the corresponding author on request.

## CRediT authorship contribution statement

**Tesfaye Lishan:** Conceptualization, Data curation, Formal analysis, Investigation, Methodology, Software, Supervision, Validation, Writing - original draft, Writing - review & editing. **Fessha Alemu:** Data curation, Investigation, Methodology, Supervision, Validation, Writing - review & editing.

## Declaration of competing interest

The authors declare that they have no known competing financial interests or personal relationships that could have appeared to influence the work reported in this paper.

## References

[1] Garcia C., Hernandez T., Coll M., Ondono S. (2017). Organic amendments for soil restoration in arid and semiarid areas: a review. AIMS Environ. Sci..

[2] Laik R., Singh S.K., Pramanick B., Kumari V., Nath D., Dessoky E.S., Hossain A. (2021). Improved method of boron fertilization in rice (Oryza sativa L.)–mustard (Brassica juncea L.) cropping system in upland calcareous soils. Sustainability.

[3] Dharminder, Singh R.K., Kumar V., Pramanick B., Alsanie W.F., Gaber A., Hossain A. (2021). The use of municipal solid waste compost in combination with proper irrigation scheduling influences the productivity, microbial activity and water use efficiency of direct seeded rice. Agriculture.

[4] Agegnehu G., Bass A., Nelson P., Bird M. (2016). Benefits of biochar, compost and biochar-compost for soil quality, maize yield and greenhouse gas emissions in a tropical agricultural soil. Sci. Total Environ..

[5] Bedada W., Karltun E., Lemenih M., Tolera M. (2014). Long-term addition of compost and NP fertilizer increases crop yield and improves soil quality in experiments on smallholder farms. Agric. Ecosyst. Environ..

[6] Ozlu E., Kumar S. (2018). Response of soil organic carbon, pH, electrical conductivity, and water stable aggregates to long-term annual manure and inorganic fertilizer. Soil Sci. Soc. Am. J..

[7] Ethio-SIS (Ethiopian Soil Information System) (2014).

[8] Cai Z., Wang B., Xu M., Zhang H., He X., Zhang L., Gao S. (2015). Intensified soil acidification from chemical N fertilization and prevention by manure in an 18-year field experiment in the red soil of southern China. J. Soils Sediments.

[9] Bhatacharyya R., Kundu S., Prakash V., Gupta H.S. (2008). Sustainability under Combined Application of Mineral and Organic Fertilizers in a Rain-Fed Soybean-Wheat System of the Indian Himalayas,” European Journal of Agronomy.

[10] Gautam Asmita (2019). https://openprairie.sdstate.edu/etd/3648.

[11] Abdelhafez A.A., Abbas M.H., Attia T.M., El Bably W., Mahrous S.E. (2018). Mineralization of organic carbon and nitrogen in semi-arid soils under organic and inorganic fertilization. Environ. Technol. Innovat..

[12] Are M., Kaart T., Selge A., Astover A., Reintam E. (2018). The interaction of soil aggregate stability with other soil properties as influenced by manure and nitrogen fertilization. Zemdirbyste-Agriculture.

[13] Von Wandruszka R. (2006). Phosphorus retention in calcareous soils and the effect of organic matter on its mobility. Geochem. Trans..

[14] Lemanowicz J., Siwik-Ziomek A., Koper J. (2014). Effects of farmyard manure and nitrogen fertilizers on mobility of phosphorus and sulphur in wheat and activity of selected hydrolases in soil. Int. Agrophys..

[15] Loveland P., Webb (2003). Is there a critical level of organic matter in the agricultural soils of temperate regions: a review. Soil Till. Res..

[16] Brady N.C. (1990).

[17] Masmoudi S., Magdich S., Rigane H., Medhioub K., Rebai A., Ammar E. (2018). Effects of compost and manure application rate on the soil physico-chemical layers properties and plant productivity. Waste Biomass. Valori..

[18] Aggelides S.M., A Londra P. (2000). Effects of compost produced from town wastes and sewage sludge on the physical properties of a loamy and clay soil. Bioresour. Technol..

[19] Oueriemmi H., Kidd P.S., Trasar-Cepeda C., Rodríguez-Garrido B., Zoghlami R.I., Ardhaoui K., Prieto-Fernández Á., Moussa M. (2021). Evaluation of composted organic wastes and farmyard manure for improving fertility of poor sandy soils in arid regions. Agriculture.

[20] Shiyam J.O., Binang W.B., Stephen G.E. (2016). Effect of animal manure on Soil nutrients replenishment and performance of Okra (Abelmoschuse Esculentus L.) grown on degraded sandy soil in Calabar. Nigeria,” Journal of Environmental.

[21] Aziz T., Ullah S., Sattar A., Nasim M., Farooq M., Khan M.M. (2010). Nutrient availability and maize (Zea mays L.) growth in soil amended with organic manures. Int. J. Agric. Biol..

[22] Ozlu E., Sandhu S.S., Kumar S., Arriaga F.J. (2019). Soil health indicators impacted by long-term cattle manure and inorganic fertilizer application in a cornsoybean rotation of South Dakota. Sci. Rep..

[23] Saidia P. (2013). Response of upland rice to activated effective microorganisms (EMA), farmyard manure and nitrogen at Ukiriguru Mwanza Tanzania. Dissertation submitted in partial fulfilment of the degree of MSC in Crop Science at Sokoine University of Agriculture, Morogoro Tanzania.

[24] Tewolde B. (2020). Gebreyohannes G.,Kidanemariam AbrhaValidation of blended NPSB fertilizer rates on yield and yield components of teff at vertisols of Hatsebo, central Tigray, Ethiopia. J. Soil Sci. Environ. Manag..

[25] (2020). Mengistu DechasaEffects of Blended Npsb Fertilizer Rates on Selected Soil Properties, Yield and Yield Components of Maize (Zea Mays L.) at Banshure Kebele in Bedele District, South Western EthiopiaMsc Thesis2022Berhe Tewolde, Girmay Gebreyohannes, Kidanemariam Abrha, Validation of blended NPSB fertilizer rates on yield and yield components of teff at vertisols of Hatsebo, central Tigray, Ethiopia. Journal of soil science and environmental management.

